# Detection of rare microorganisms in bone and joint infections by metagenomic next-generation sequencing: discrimination, decision-making, and clinical benefits

**DOI:** 10.1302/2046-3758.138.BJR-2023-0420.R1

**Published:** 2024-08-15

**Authors:** Hongxin Hu, Haiqi Ding, Jianhua Lyu, Yang Chen, Changyu Huang, Chaofan Zhang, Wenbo Li, Xinyu Fang, Wenming Zhang

**Affiliations:** 1 Department of Orthopaedics, Affiliated Hospital of Putian University, Putian, China; 2 Department of Orthopedic Surgery, The First Affiliated Hospital of Fujian Medical University, Fuzhou, China; 3 Department of Orthopaedic Surgery, National Regional Medical Center, Binhai Campus of the First Affiliated Hospital, Fujian Medical University, Fuzhou, China; 4 Fujian Provincial Institute of Orthopedics, the First Affiliated Hospital, Fujian Medical University, Fuzhou, China

**Keywords:** Rare microorganisms, Bone and joint infections, Metagenomic next-generation sequencing, joint infections, microorganisms, antibiotics, infections, clinical diagnosis, retrospective analysis, bacteria, t-test, chi- squared test, clinicians

## Abstract

**Aims:**

This aim of this study was to analyze the detection rate of rare pathogens in bone and joint infections (BJIs) using metagenomic next-generation sequencing (mNGS), and the impact of mNGS on clinical diagnosis and treatment.

**Methods:**

A retrospective analysis was conducted on 235 patients with BJIs who were treated at our hospital between January 2015 and December 2021. Patients were divided into the no-mNGS group (microbial culture only) and the mNGS group (mNGS testing and microbial culture) based on whether mNGS testing was used or not.

**Results:**

A total of 147 patients were included in the no-mNGS group and 88 in the mNGS group. The mNGS group had a higher detection rate of rare pathogens than the no-mNGS group (21.6% vs 10.2%, p = 0.016). However, the mNGS group had lower rates of antibiotic-related complications, shorter hospital stays, and higher infection control rates compared with the no-mNGS group (p = 0.017, p = 0.003, and p = 0.028, respectively), while there was no significant difference in the duration of antibiotic use (p = 0.957). In culture-negative cases, the mNGS group had lower rates of antibiotic-related complications, shorter hospital stays, and a higher infection control rate than the no-mNGS group (p = 0.036, p = 0.033, p = 0.022, respectively), while there was no significant difference in the duration of antibiotic use (p = 0.748).

**Conclusion:**

mNGS improves detection of rare pathogens in BJIs. mNGS testing reduces antibiotic-related complications, shortens hospital stay and antibiotic use duration, and improves treatment success rate, benefits which are particularly evident in culture-negative cases.

Cite this article: *Bone Joint Res* 2024;13(8):401–410.

## Article focus

- This study aimed to analyze the detection of rare bacteria in bone and joint infections (BJIs) by metagenomic next-generation sequencing (mNGS) and its impact on clinical diagnosis and treatment.

## Key messages

- The use of mNGS can enhance the detection rate of rare pathogens in BJIs, shorten the length of hospital stays, reduce antibiotic-related complications, and improve the infection control rate.
- For cases that are culture-negative or show poor response to treatment despite positive cultures, mNGS represents a viable option.

## Strengths and limitations

- This study stands out as one of the few clinical investigations directly addressing rare bacteria.
- However, it is essential to acknowledge that this was a single-centre retrospective study.
- There is a need for a multicentre study to comprehensively assess the diagnostic value of mNGS in detecting rare bacteria in BJIs.

## Introduction

A rising trend has been observed in bone and joint infections (BJIs), including primary or secondary osteomyelitis, septic arthritis, and implant-related infections.^1^ The majority of causative microorganisms are staphylococci, streptococci, and gram-negative bacteria.^2-4^ However, the incidence of BJIs is on the rise due to factors such as ageing, obesity, the use of immunosuppressive drugs, invasive procedures, and endosurgery.^5^ Notably, there is a growing presence of less frequently detected microorganisms, including *Mycoplasma* and non-tuberculous mycobacteria (NTM). Consequently, diagnosing and treating BJIs caused by these microorganisms continues to pose a considerable challenge in clinical practice.^6-8^

Bacterial culture is the gold standard for diagnosing BJIs and guiding the application of antibiotic regimens.^9-11^ However, in clinical practice, bacterial cultures have been found to be negative despite the presence of suppuration, sinus tracts, elevated inflammatory markers, or histopathological signs of infection.^12^ It has been noted that cultures remain negative in 5% to 42% of cases, even though there were other indicators of infection.^13,14^ This phenomenon is attributed to inadequate culture time, pre-culture administration of antibiotics, and substandard clinical sampling procedures.^1,15^ Additionally, infections caused by other pathogens contribute to these phenomena. Previous reports categorize these pathogens as “uncommon”, “atypical”, “unusual”, and “rare” microorganisms;^8,16,17^ we use the term “rare microorganisms” and will do so throughout this article. Most rare microorganisms, such as *Mycoplasma*, NTM, and *Parvimonas micra*, require special culture conditions and are not detectable by conventional clinical microbiology laboratories.^1,18,19^

The advancement of molecular diagnostic techniques has enhanced the detection of rare microorganisms. While predefined targets, such as specific primers or probes, in polymerase chain reaction (PCR) enable the detection of rare microorganisms with specific sequences, PCR cannot detect pathogens beyond the defined target.^20,21^ Metagenomic next-generation sequencing (mNGS) provides a comprehensive screening approach through unbiased sampling of collected tissue samples, followed by high-throughput sequencing and bioinformatics comparison. This encompasses various microorganisms that may be present, such as bacteria, fungi, viruses, and parasites.^10,22^ This advancement has enabled the clinical detection of rare microorganisms, and has been widely employed for infectious disease diagnosis in many fields, with numerous reports documenting the detection of these microorganisms in cases of infection.^22,23^

Since 2017, mNGS has been used at our centre (The First Affiliated Hospital of Fujian Medical University) to aid in the diagnosis of BJIs, leading to the identification of several rare microorganisms.^22,23^ A crucial question revolves around determining whether these detected rare microorganisms are indeed the primary causative agents of BJIs. Another challenge lies in acquiring drug susceptibility-related information from the test results, primarily due to the genetic complexity of the mNGS test samples. Consequently, applying the test results to guidance on clinical drug usage continues to pose difficulties.

With this in mind, we analyzed the BJIs treated at our centre over the past seven years, including cases with and without mNGS testing. We analyzed the detection of rare microorganisms and the impact of mNGS detection on the clinical process. Our aim was to determine whether mNGS can provide meaningful assistance to clinicians in terms of cost-effectiveness and patient benefit, and to identify which cases of BJIs were most suitable for the application of mNGS.

## Methods

### Patient selection and clinical data collection

This study included patients with BJIs treated at our centre between 2015 and 2021, and all diagnoses were determined by two senior orthopaedic surgeons (WZ, WL), two senior microbiologists, and two senior infection specialists. BJIs as defined in this study included the following types: 1) primary septic arthritis; 2) periprosthetic joint infections (PJIs), including infections that persisted after Stage I and Stage II revision, and debridement, antibiotics and implant retention (DAIR); 3) implant-related infections; 4) osteomyelitis; and 5) haematogenous infections (such as those caused by invasive dental procedures, periodontitis, or indwelling catheters). Inclusion criteria were as follows: 1) patients diagnosed with BJIs who met the diagnostic criteria;^24,25^ 2) patients with complete medical record data; and 3) patients who agreed to participate in this study. Exclusion criteria were as follows: 1) insufficient specimens were obtained to set up bacterial cultures and/or mNGS; 2) contamination or suspected contamination of samples. The group with bacterial culture alone for BJIs was included in the group without mNGS testing (no-mNGS group); those with both bacterial culture and mNGS testing were included in the mNGS group (mNGS group). The demographic characteristics, microbiological culture results, treatment regimen, medication regimen, complications, length of hospital stay, and infection control were recorded for all enrolled patients, and for those who underwent mNGS testing, the mNGS test results were recorded. This retrospective study was approved by our hospital ethics committee.

### Definition and criteria of bone and joint infections caused by rare microorganisms

**Fig. 1:**
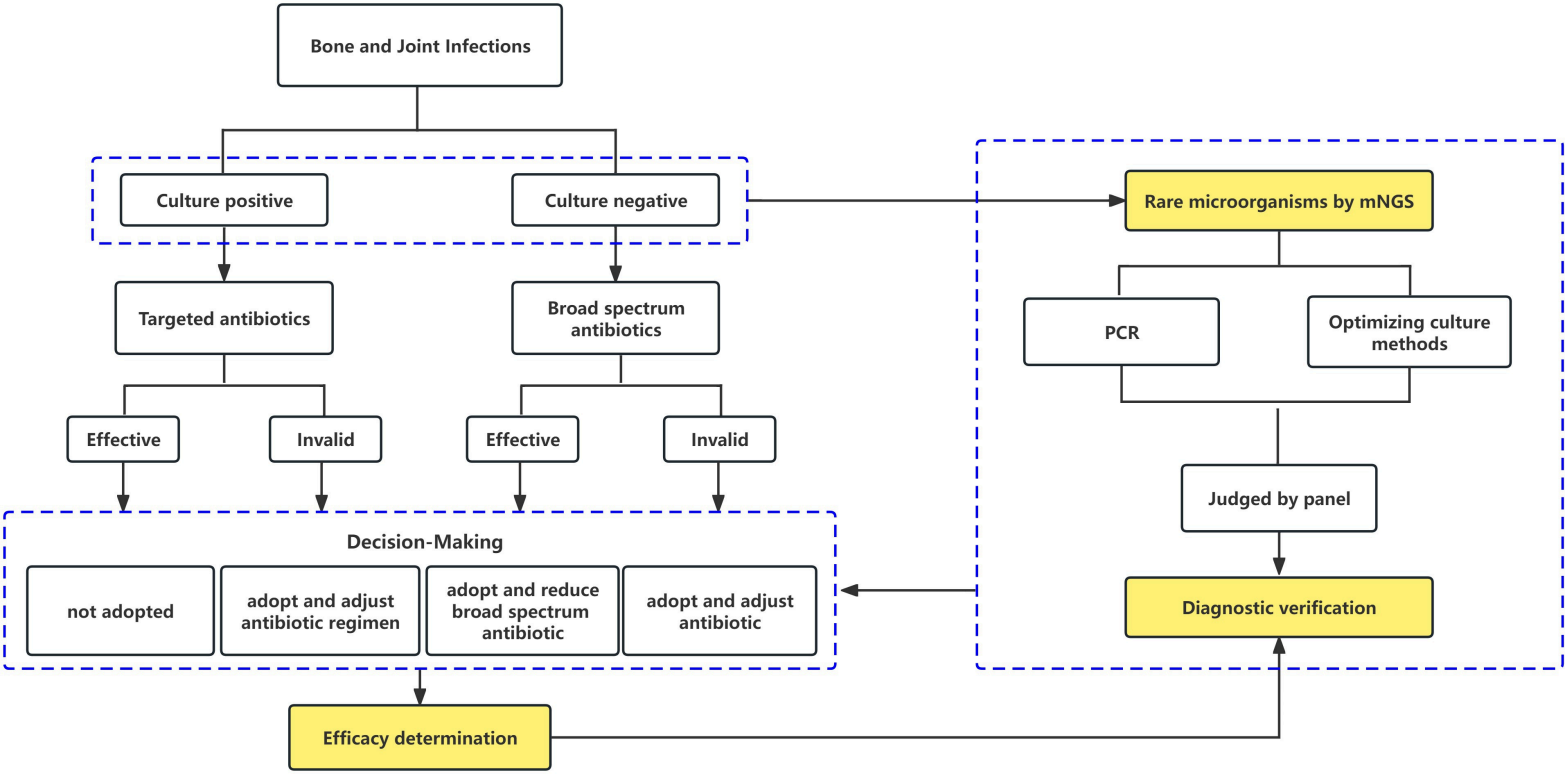
Diagnosis process of rare microorganisms and guidance of clinical treatment. mNGS, metagenomic next-generation sequencing; PCR, polymerase chain reaction.

There is no clear definition of BJIs caused by rare microorganisms, which can lead to clinical misinterpretation due to over-diagnosis. This article presents criteria established by a team of microbiologists, infectious disease specialists, and orthopaedic surgeons for the definitive identification of BJIs caused by rare microorganisms: 1) the technical specifications of the microorganisms detected by mNGS meet the criteria (for the mNGS interpretation below); 2) clinicians and microbiologists jointly confirm that the pathogen caused an infection in the bone and joint or other organs and had been previously reported in the literature; 3) the pathogen is not typically associated with BJIs, or it was described in a maximum of ten case reports or a small case series about BJIs; 4) based on NGS results, there were indications of a potential infection caused by rare microorganisms and subsequently, cultivation conditions were adjusted specifically for the rare microorganisms, leading to successful cultivation and subsequent identification (Figure 1); and 5) if all experts agreed to change the anti-infective regimen based on the pathogen diagnosis from mNGS, but treatment was ineffective, the pathogen diagnosis would be considered a misdiagnosis (Figure 1).

### Specimen collection and microbiological culture

After collection, the specimens were transferred to the microbiology room within 30 minutes for the subsequent microbial culture process. In order to improve the positive rate of microbial culture, we adhered to a specific culture protocol, as outlined below.

### Fluid specimens

Fluid specimens (e.g. synovial fluid, pus) were extracted and injected into Bactec Plus/F aerobic and anaerobic blood culture bottles (BD, Germany) or BactecPeds Plus/F blood culture bottles (BD) and then placed in a Bactec 9050 automated thermostat (BD) for 14 days.

### Tissue specimens

Tissue specimens were placed in sterile EP grinding tubes with 1 ml of brain heart infusion broth (Qingdao Haibo Biotechnology, China) and placed in an automatic rapid grinder (Shanghai Jingxin Industrial Development, China, JXFSTPRP-24) for homogenization (40 Hz, 60 to 90 s). The homogenized tissue specimens were then inoculated onto blood agar plates (Thermo Fisher Scientific, USA), and cultured under aerobic and anaerobic conditions for 14 days.

Intraoperatively removed implants (such as prosthesis or plates) were placed in sterilized containers and submerged in sterile saline. Next, the containers were placed under ultrasonication (40 Hz, 5 mins) to disrupt the biofilm on the implant surface, followed by centrifugation for five minutes (4,000 r/min) and discarding the supernatant. The implants were then resuspended with sterile saline and injected into Bactec Plus/F culture flasks and Bactec Lytic/10/F culture flasks in a Bactec 9050 autostart (BD) for a 14-day culture period. The mNGS procedure and the interpretation of mNGS are provided in the Supplementary Material.

### Optimization of culture based on mNGS results

The collected specimens underwent the aforementioned pre-treatment procedure before undergoing mNGS testing. The mNGS results were meticulously analyzed to determine suitable culture conditions and duration, tailored to the microbial species suggested by mNGS, especially rare bacteria.^23^

### Outcome evaluation

Success in infection control was evaluated based on the 2013 international consensus,^26^ which encompassed the following criteria: 1) eradication of infection, defined as good wound healing with the absence of sinus tracts, exudation, return to normal levels of serological markers, no need for antibiotics, as well as no recurrence of infection caused by the same bacteria; 2) no subsequent operative intervention due to infection; and 3) no infection-related death (caused by sepsis, necrotizing fasciitis, etc).

Antibiotic-related complications included: 1) myelosuppression, as characterized by a leucocyte count > 4 × 10^9^/l before the use of antibiotics and a leucocyte maximum < 3 × 10^9^/l during antibiotic treatment; 2) hepatic impairment, as indicated by normal levels of alanine aminotransferase (ALT) and aspartate aminotransferase (AST) before using antibiotics, and the peak value of ALT or AST increasing more than 1.5 times during the use of antibiotics; 3) renal impairment, as marked by normal creatinine levels before using antibiotics, and creatinine values that increased more than 1.5 times during antibiotic treatment; and 4) gastrointestinal symptoms.^27^

### Demographic characteristics

Table I presents the general demographic characteristics of the no-mNGS and mNGS groups in this study. The no-mNGS group consisted of 163 patients, with seven excluded due to absence of microbial culture and nine due to incomplete medical data, resulting in 147 patients included for analysis. The mean age of the no-mNGS group was 61.75 years (standard deviation (SD) 12.09), comprising 74 males and 73 females. Among these patients, 89 had PJI, 38 had primary septic arthritis, 15 had osteomyelitis, and five had implant-related infections. The mNGS group comprised 111 patients, with nine not undergoing microbial culture and 14 unable to provide complete medical data; thus, 88 patients were included in the final analysis. The mean age of the mNGS group was 63.81 years (SD 13.73), with 44 males and 44 females. In the mNGS group, 61 patients had PJI, 19 had primary septic arthritis, five had osteomyelitis, and three had implant-related infections.

**Table I.** Demographic characteristics of all patients.

Variable	No-mNGS group (n = 147)	mNGS group (n = 88)	Statistical value	p-value
Sex (male/female), n	74/73	44/44	0.003*	0.960
Median age, yrs (IQR)	63.00 (55.00 to 69.00)	67.00 (57.00 to 71.00)	-1.679†	0.093
Mean BMI, kg/m^2^ (SD)	24.08 (3.23)	24.89 (3.26)	-1.840‡	0.067
Hypertension, n	41	30	1.003*	0.316
Diabetes, n	27	15	0.066*	0.798
Sinus, n	34	26	1.192*	0.275
Primary septic arthritis, n	38	19	0.544*	0.461
PJI, n	89	61	1.835*	0.175
Implant-related infection, n	5	3	0.000*	0.997
Osteomyelitis, n	15	5	1.446*	0.229
Administration of pre-diagnosis antibiotics, n	49	39	2.836*	0.092

* Chi-squared test.

† Mann-Whitney U test.

‡ Independent-samples *t*-test.

mNGS, metagenomic next-generation sequencing; PJI, periprosthetic joint infection.

### Statistical analysis

Demographic characteristics and comorbidities between the two groups were compared using the chi-squared test, independent-samples *t*-test, or Mann-Whitney U test. Continuous variables were presented as mean and SD, and analyzed with independent-samples *t*-test if they followed a normal distribution. If they did not follow a normal distribution, the Mann-Whitney U test was used and expressed as medians (interquartile ranges (IQRs)). The chi-squared test was used to compare the incidence of antibiotic complications. Kaplan-Meier survival analysis and the log-rank method were used to assess infection control. All statistical analyses were conducted using SPSS v20.0 (IBM, USA), with statistical significance set at p < 0.05.

## Results

### Overview of the identified organisms

As shown in Table II, staphylococcus is the predominant pathogenic bacteria in the no-mNGS group (49.0%), with *S. aureus* (26.5%) and *S. epidermidis* (10.9%). Likewise, staphylococcus remains prevalent in the mNGS group (37.5%), with *S. aureus* accounting for 20.5% and *S. epidermidis* accounting for 13.6%. The distribution of all pathogens was presented in Table II. The positive rate of microbial culture in the no-mNGS group was approximately 64.6% (95/147), with eight cases of polymicrobial infections. The positive rate in the mNGS group was 78.4% (69/88), with six cases of polymicrobial infections. Use of mNGS notably enhanced the detection rate of microorganisms (p = 0.026, chi-squared test). Moreover, the detection rate of rare microorganisms in the no-mNGS group was 10.2% (15/147), while in the mNGS group it was 21.6% (19/88). These findings demonstrate a significant enhancement in the detection rate of rare microorganisms with mNGS (p = 0.016, chi-squared test).

**Table II.** Overview of the organisms identified in both groups.

Organism	No-mNGS group, n (%)	mNGS group, n (%)
*Staphylococcus aureus*	39 (26.5)	18 (20.5)
MSSA	21 (14.3)	7 (8.0)
MRSA	18 (12.2)	11 (12.5)
*Staphylococcus epidermidis*	16 (10.9)	12 (13.6)
*Staphylococcus capitis*	5 (3.4)	1 (1.1)
*Escherichia coli*	5 (3.4)	3 (3.4)
*Pseudomonas aeruginosa*	5 (3.4)	4 (4.5)
*Staphylococcus haemolyticus*	3 (2.0)	1 (1.1)
*Streptococcus haemolyticus*	0 (0)	1 (1.1)
*Enterococcus faecalis*	0 (0)	7 (8.0)
*Staphylococcus hominis*	3 (2.0)	0 (0)
*Enterobacter cloacae*	2 (1.4)	2 (2.3)
*Klebsiella pneumoniae*	3 (2.0)	0 (0)
*Burkholderia cepacia*	0 (0)	1 (1.1)
*Staphylococcus lugdunensis*	3 (2.0)	1 (1.1)
*Finegoldia magna*	1 (0.7)	1 (1.1)
*Pseudomonas stutzeri*	0 (0)	1 (1.1)
*Micrococcus luteus*	0 (0)	1 (1.1)
*Acinetobacter baumannii*	2 (1.4)	1 (1.1)
*Candida albicans*	1 (0.7)	2 (2.3)
*Streptococcus agalactiae*	0 (0)	3 (3.4)
*Morganella morganii*	1 (0.7)	0 (0)
*Candida parapsilosis*	0 (0)	2 (2.3)
*Salmonella gallisepticum serotype*	2 (1.4)	0 (0)
*Mycobacteroides abscessus*	0 (0 )	1 (1.4)
*Enterobacter cloacae*	2 (1.4)	2 (2.3)
*Enterococcus gallinarum*	0 (0)	1 (1.1)
*Streptococcus dysgalactiae subsp. Equine*	2 (1.4)	1 (1.1)
*Mycobacterium fortuitum*	0 (0)	2 (2.3)
*Nocardia farcinica*	1 (0.7)	0 (0)
*Ralstonia pickettii*	1 (0.7)	0 (0)
*Bacteroides fragilis*	1 (0.7)	0 (0)
*Propionibacterium acnes*	1 (0.7)	0 (0)
*Staphylococcus warneri*	2 (1.4)	0 (0)
*Aggregatibacter aphrophilus*	1 (0.7)	0 (0)
*Streptococcus equisimilis*	1 (0.7)	0 (0)
*Stenotrophomonas maltophilia*	0 (0)	1 (1.1)
*Pseudomonas monteilii*	0 (0)	1 (1.1)
*Streptococcus oralis*	0 (0)	1 (1.1)
*Helcococcus kunzii*	0 (0)	1 (1.1)
*Staphylococcus pasteuri*	1 (0.7)	0 (0)
*Serratia marcescens*	1 (0.7)	0 (0)
*Salmonella enterica subsp. enterica*	1 (0.7)	0 (0)
*Mycobacterium ulcerans*	0 (0)	1 (1.1)
*Mycobacterium colombiense*	0 (0)	1 (1.1)
Multiple infections	8 (5.4)	6 (6.8)
Culture-negative	52 (35.4)	19 (21.6)

mNGS, metagenomic next-generation sequencing; MRSA, methicillin-resistant *S. aureus*; MSSA, methicillin-susceptible *S. aureus*.

### Comparison of therapeutic effects between the two groups

To investigate the impact of mNGS testing on the treatment of BJIs, we compared treatment efficacy between the groups using mNGS and those not using mNGS (Table III). Results indicated that after implementation of mNGS testing, differences in antibiotic treatment regimens were observed, primarily in other anti-infection regimens (Table III). Furthermore, no significant differences were found in the duration of antibiotic usage between the two groups with or without the mNGS (p = 0.957, independent-samples *t*-test). Nevertheless, the introduction of mNGS resulted in a significant reduction in antibiotic-related complications (p = 0.017, chi- squared test) and length of stay in the mNGS group compared with the no-mNGS group (p = 0.026, Mann-Whitney U test).

**Table III.** Comparison of clinical efficacy between the groups.

Variable	No-mNGS group (n = 147)	mNGS group (n = 88)	Statistical value	p-value
Mean time of antibiotic use, days (SD)	87.31 (29.72)	87.06 (37.52)	-0.055*	0.957
**Antibiotic regimen, n (%)**			16.315†	0.001
Vancomycin + other	108 (73.5)	58 (65.9)	1.517†	0.218
Fluoroquinolone + other	15 (10.2)	2 (2.3)	5.160†	0.023
Cefuroxime/Cefazolin	14 (9.5)	8 (9.0)	0.012†	0.912
Other	9 (6.1)	19 (21.6)	12.550†	0.000
Complications, n (%)	31 (21.1)	8 (9.1)	5.724†	0.017
Median length of stay, days (IQR)	22.00 (15.00 to 27.00)	19.00 (15.00 to 23.00)	-2.224‡	0.026

* Independent-samples *t*-test.

† Chi-squared test.

‡ Mann-Whitney U test.

mNGS, metagenomic next-generation sequencing.

**Fig. 2:**
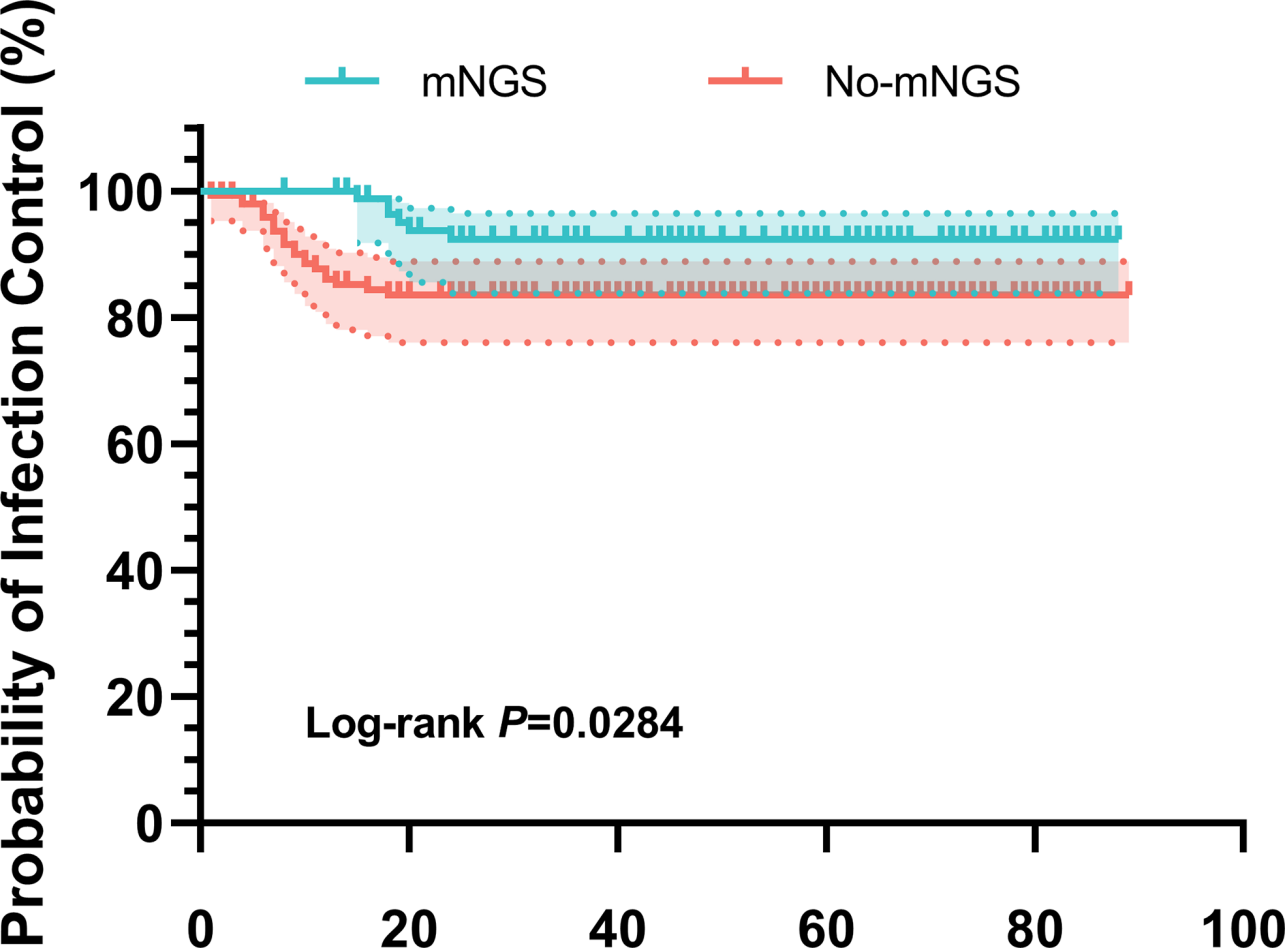
Kaplan-Meier survival curve analysis results of the two groups. The shaded areas denote 95% CIs. mNGS, metagenomic next-generation sequencing.

Kaplan-Meier survival curves were plotted for the no-mNGS group and the mNGS group, and the log-rank method was used for comparison. Results showed that the mNGS group exhibited an advantage in infection control (p = 0.028, log-rank test) (Figure 2).

### Comparison of therapeutic effects between two groups of culture-negative cases

To further demonstrate the impact of mNGS on culture-negative BJIs, we compared the effectiveness of treatment between the two groups. Table IV reveals that in culture-negative cases, there was no significant difference in the duration of antibiotic administration between the two groups (p = 0.748, independent-samples *t*-test); however, the mNGS group exhibited a shorter length of stay (p = 0.033, Mann-Whitney U test). Regarding the choice of antibiotic regimen, there was no significant difference between the two groups in the vancomycin, quinolones, or cefazolin/cefuroxime regimens, except for other antibiotic regimens (Table IV). With regard to treatment efficacy for culture-negative patients in both groups, the mNGS group exhibited notably fewer antibiotic-related complications compared to the no-mNGS group.

**Table IV.** Comparison of clinical efficacy between two groups with negative culture.

Variable	No-mNGS group (n = 52)	mNGS group (n = 19)	Statistical value	p-value
Mean time of antibiotic use, days (SD)	83.90 (23.93)	86.54 (36.38)	-0.324*	0.748
**Antibiotic regimen, n (%)**			9.299†	0.018
Vancomycin + other	31 (59.6)	8 (54.1)	1.723‡	0.189
Fluoroquinolone + other	8 (15.4)	0 (0)	1.935§	0.164
Cefuroxime/Cefazolin	8 (15.4)	4 (16.7)	0.043§	0.836
Other	5 (9.6)	7 (29.2)	5.534§	0.019
Complications, n (%)	18 (34.6)	1 (5.3)	4.386§	0.036
Median length of stay, days (IQR)	19.50 (13.00 to 25.00)	15.00 (11.00 to 20.00)	-2.127¶	0.033

* Independent-samples *t*-test.

† Fisher’s exact test.

‡ Chi-squared test.

§ Continuity correction of chi-squared test.

¶ Mann-Whitney U test.

mNGS, metagenomic next-generation sequencing.

**Fig. 3:**
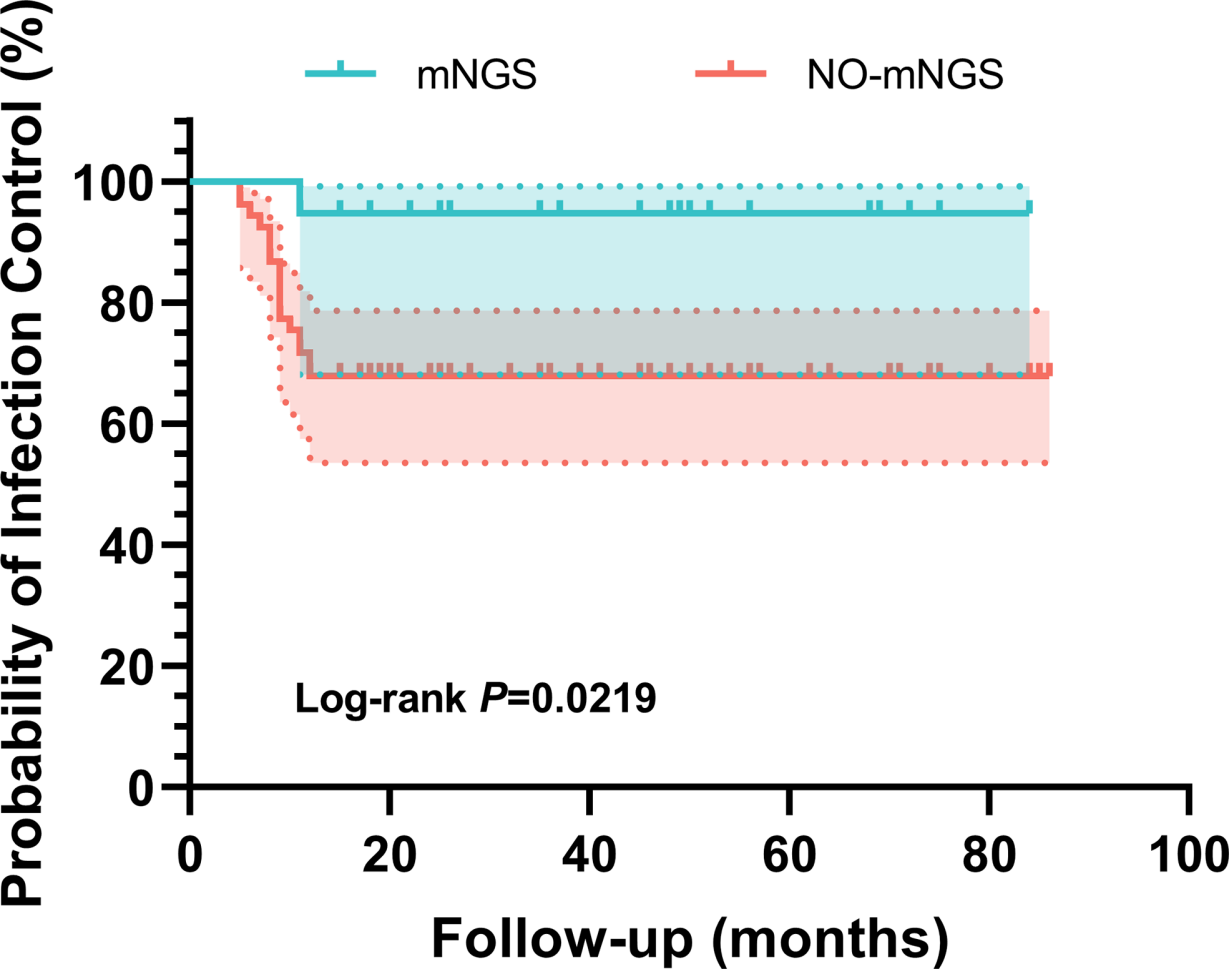
Kaplan-Meier survival curve analysis results of culture-negative patients in both groups. The shaded areas denote 95% CIs. mGNS, metagenomic next-generation sequencing.

Additionally, Kaplan-Meier survival curves were plotted for culture-negative patients in both groups, and their comparison was conducted using the log-rank method. The mNGS group demonstrated superiority in controlling culture-negative infection (p = 0.022, log-rank test) (Figure 3).

Typical cases of BJIs caused by rare organisms accurately diagnosed by mNGS are provided in the Supplementary Material.

## Discussion

The advancement of mNGS aids in diagnosing rare microorganisms, however the incidence of BJIs caused by rare bacteria remains undefined. Previous reports in the literature indicate that rare bacteria constitute 5% to 10% of BJIs,^8,28^ based on microbial culture results. In our study, we observed a comparable proportion (10.2%) of rare microorganisms identified through microbial culture alone. Utilizing mNGS boosts the detection rate of rare microorganisms to 21.6% due to its high throughput and advanced databases. Rigorous identification processes and targeted drug therapy mitigate antibiotic-related side effects and enhance efficacy.

Distinguishing rare bacteria identified by mNGS from pathogenic, background, or commensal bacteria is crucial to prevent over-interpretation of test results and diagnostic bias, and therefore overuse of antibiotics to treat bacteria that are not pathogenic. Advances in surgical procedures, sample collection, and diagnostic techniques have facilitated the detection of more rare bacteria. To mitigate this challenge, we implemented a rigorous process for interpreting mNGS results concerning rare bacteria (Figure 1). PCR methods are commonly employed to validate pathogens identified by mNGS; we refined culture based on mNGS results to enhance the positive rate of culturing pathogenic bacteria.^23^ For instance, in Case 1, although preoperative and intraoperative culture yielded negative results, mNGS indicated the presence of *Mycobacterium houstonense*. In conjunction with the mNGS results, we used intraoperative retention tissue and subjected it to mycobacteria testing using the BACTEC MGIT system for four days, resulting in the growth and identification of the strain as *Mycobacterium houstonense*. This approach not only confirmed the causative organism, but also addressed the issue of molecular diagnosis without drug susceptibility testing. The accuracy of diagnosing rare bacteria was validated based on the final treatment efficacy, and all instances of rare bacteria cited in this paper were diagnosed and confirmed to be effectively treated.

When the pathogen remains unidentified, clinicians typically resort to broad-spectrum antibiotics and then adjust the antibiotic regimen once the pathogen is identified.^21^ In instances of culture-negative infections, failure to detect the pathogen can result in the overuse of broad-spectrum antibiotics, an increased risk of drug resistance, and escalated healthcare costs.^10,29^ Consequently, mNGS testing offers insights into potential pathogens, proving beneficial for culture-negative BJIs. First, when empirical broad-spectrum antibiotics prove effective, mNGS results serve as a foundation for antibiotics usage, reducing the misuse of antibiotics and associated side effects. In our study, the culture-negative mNGS group exhibited significantly fewer antibiotic side effects and a higher treatment success rate compared to the culture-negative group without mNGS. For example, in culture-negative Case 2, the patient received vancomycin and ceftazidime as broad-spectrum antibiotics. However, upon integrating mNGS results, *Parvimonas micra* was identified as the pathogenic bacteria, prompting treatment adjustment to amoxicillin, resulting in eventual recovery. *Parvimonas micra*, although challenging to diagnose, was found to be susceptible to penicillin and clindamycin.^30^ Even when not cultured, routine treatment regimens typically include the broad-spectrum antibiotic penicillin. Second, in cases where empirical treatment proves ineffective, adjusting antibiotics based on mNGS results leads to successful treatment. For example, in Case 3, initial treatment with vancomycin and meropenem failed to control the infection, but adjusting the antibiotic regimen to pyrazinamide, isoniazid, rifampin, and ethambutol based on the mNGS results proved effective in controlling the infection. Similarly, in Case 4, empirical treatment with vancomycin and meropenem did not suffice, but upon changing to clarithromycin, ethambutol, and rifampin based on mNGS results, the infection was controlled, indicating the presence of *Mycobacterium colombiense* infection. One previous study demonstrated that culture-negative BJIs often lead to inefficacy due to the absence of pathogenic information.^15^ This underscores the importance of pathogen identification for successful antibiotic therapy.

In cases with positive microbial cultures, mNGS can detect additional potentially pathogenic organisms. According to our treatment protocol, we do not advocate adding antibiotics based on mNGS results if targeted drug therapy derived from microbial culture results is effective. However, we recommend adjusting antibiotics based on mNGS results when targeted therapy proves ineffective. For instance, in Case 5, although the microbiological culture suggested *S. aureus*, treatment with vancomycin and meropenem was ineffective. Subsequently, considering the mNGS result indicating *Mycoplasma hominis* infection, treatment with doxycycline proved successful in controlling the infection.

Some studies have emphasized the necessity for targeted drugs. However, rare microorganisms may evade coverage by broad-spectrum antibiotics like vancomycin and meropenem, potentially leading to resistance.^31,32^ For example, *Mycoplasma* lacks cell walls, rendering them impervious to conventional empirical antibiotics such as vancomycin and β-lactam antibiotics; instead, specific drugs like tetracyclines or fluoroquinolones are recommended.^1,31,33^ Similarly, NTM infections often necessitate prolonged therapy with anti-mycobacterial drugs and surgical intervention, extending up to six to 12 months in severe cases.^32,34^ Rare microorganisms pose challenges in detection, often prompting the use of empirical broad-spectrum antibiotics before identification, while targeted drugs are employed afterwards, thereby prolonging the time to antibiotic administration. Hence, there was no discrepancy in the duration of antibiotic treatment between the two groups in this study. To enhance treatment effectiveness, we recommend utilizing mNGS for screening in cases of culture-negative BJIs to aid in pathogen detection using mNGS, and in cases of culture-positive BJIs with inadequate treatment outcomes to identify additional pathogens using mNGS that may contribute to the suboptimal treatment response.

Patients with autoimmune diseases, hypogammaglobulinemia, other underlying immunosuppression, and recent history of invasive urogenital tract manipulation face a heightened risk for BJIs caused by rare microorganisms.^1,17^ At our institution, preoperative synovial fluid mNGS testing serves as a supplementary diagnostic tool for such patients, offering valuable reference information for physicians.^23^ Intraoperatively obtained synovial fluid, additional tissue, or ultrasound lysate samples can be used to corroborate mNGS results through various methods, including optimized microbial culture techniques and DNA extraction followed by 16S PCR amplification and sequencing. A panel assesses these findings to determine whether adjustment of the antibiotic regimen is warranted based on the effectiveness of the original antibiotic treatment for the detected rare bacterial infection. Ultimately, the final diagnosis hinges on the treatment’s efficacy (Figure 1).

Over-interpretation of mNGS results for rare bacterial tests may result in misdiagnosis and unnecessary antibiotic treatment, necessitating advanced techniques and seasoned physicians to tackle this challenge. Moreover, the present study has some limitations. First, it is retrospective, introducing potential selection bias regarding the decision to opt for mNGS testing and subsequent treatment based on mNGS results, which could impact the findings. Second, variations in specimens used for mNGS testing may influence the results due to the differing detection capabilities of mNGS across various tissues. Third, culturing rare bacteria poses challenges, and defining positive culture based on at least two specimens may lead to overlooking some rare bacteria. Lastly, future multicentre studies are warranted to validate the diagnostic and therapeutic efficacy of mNGS for rare bacteria.

Adjusting culture conditions based on mNGS results enhances the rate of positive cultures for rare bacteria in BJIs. Utilizing mNGS testing to treat BJIs, particularly in culture-negative cases, can significantly reduce antibiotic-related complications, shorten hospital stays, and improve treatment success rates. In cases of BJI, a rigorous identification and validation method should be employed to confirm the rare bacteria detected by mNGS as true pathogenic bacteria. The identification of rare pathogenic bacteria through a rigorous identification process can provide clinicians with guidance for diagnosis and treatment, ultimately enhancing the cure rate.

## Data Availability

The data that support the findings for this study are available to other researchers from the corresponding author upon reasonable request.
